# Evaluation of probiotic potential, safety assessment and whole genome sequencing of *Lactiplantibacillus plantarum* strain MOVIN isolated from toddy sample

**DOI:** 10.3389/fmicb.2025.1625659

**Published:** 2025-07-10

**Authors:** A. Vijayaganapathi, V. Mohanasrinivasan

**Affiliations:** School of Bio Sciences and Technology, Vellore Institute of Technology, Vellore, India

**Keywords:** toddy, probiotics, lactic acid bacteria, cell surface hydrophobicity, secondary metabolites

## Abstract

**Introduction:**

Toddy, an alcoholic drink produced through natural fermentation, is a significant reservoir of probiotic microorganisms. Probiotics are increasingly studied for their beneficial effects in managing gastrointestinal disorders, enhancing immune responses, and promoting overall health. *Lactiplantibacillus plantarum* is a well-known probiotic strain as it possesses gastrointestinal survival, antimicrobial activity, and adaptability to various environmental factors. This study aims to isolate and characterize probiotic bacteria from toddy samples, with a focus on their bioactive and genomic attributes.

**Methods:**

Twenty bacterial strains were isolated from toddy samples and subjected to preliminary biochemical screening. Of these, 17 isolates were selected for further screening of antibacterial activity against foodborne pathogens. These isolates were also evaluated for probiotic traits such as auto-aggregation, hydrophobicity, tolerance to bile, acid, and NaCl. Antibiotic susceptibility was assessed against nine antibiotics. The most promising isolate was subjected to whole-genome sequencing, followed by *in silico* genomic analysis using BAGEL4, antiSMASH, and the KEGG automatic annotation server.

**Results:**

Among the 17 selected isolates, VITVTD-3, VITVTO-5, and VITVH-5 exhibited inhibitory effects against all tested food-borne pathogens. These isolates also showed moderate levels of auto-aggregation and hydrophobicity, along with the ability to withstand bile salts, acidic pH, and high salt concentrations. Antibiotic profiling revealed resistance to vancomycin in all three isolates. Antioxidant and haemolytic activity assays further supported their probiotic potential. Of these, VITVTD-3 demonstrated the most favourable combination of probiotic attributes. Whole-genome sequencing revealed that this isolate corresponds to *Lactiplantibacillus plantarum* strain MOVIN, possessing a genome size of 3.16 Mb and a GC content of 44.4%. Genomic analysis revealed the presence of a plantaricin operon and multiple bacteriocin gene clusters. KEGG annotation predicted 5,599 genes involved in 23 different metabolic pathways.

**Discussion:**

The findings confirm that *Lactiplantibacillus plantarum* MOVIN exhibits strong probiotic characteristics, supported by phenotypic, functional, and genomic evidence. The presence of bacteriocin biosynthesis genes, along with the ability to inhibit pathogenic microorganisms and survive gastrointestinal conditions, highlights its suitability for probiotic applications. Further functional studies and safety evaluations are warranted to explore its potential use in food or therapeutic formulations.

## 1 Introduction

Fermented foods and drinks which are mainly produced from cereals and fruits as a base, are highly popular all around the world (Gebre et al., 2023). One of the early biotechnological practices used by Indians is the preparation of traditional fermented foods, where bacteria are essential for improving food quality, preserving food, enriching meals, and improving health (Nath et al., 2021; Ray et al., 2016). Toddy is one of the naturally fermented alcoholic beverages consumed by people in many parts of the world, especially in India, for many years. This naturally fermented drink contains many health-beneficial values to humans and is also considered a probiotic drink (Moorthy et al., 2015).

Consumers are highly attracted to flavoured tasty foods and also, they are concerned about the health benefits, such as low-calorie and low-fat foods. This will lead to the new product development in the food industry with improved nutritional and functional qualities as well as notable sensory quality (Pino et al., 2022; Guiné et al., 2020). Hence, food based companies are more focused on incorporating food products using beneficial bacteria such as “probiotics,” which fall under the category of “functional foods” (Islam et al., 2018). Probiotics are live microorganisms that, when ingested in adequate quantities, provide multiple health benefits to the host. These beneficial effects occur through various mechanisms that support the host’s physiological functions. The consumption of these microbes in sufficient amounts is essential to achieve their positive impact on overall health (Islam et al., 2018; Vijayabaskar and Somasundaram, 2008; Jayashree et al., 2010; Joint FAO/WHO Expert Committee on Food Additives, 2002).

Over the decade, probiotics have emerged as a prominent area of study for lactic acid bacteria, with the genera *Lactobacillus* and *Bifidobacterium* receiving the greatest interest due to their potential to naturally improve human health (Magdoub et al., 2015). Probiotics are beneficial bacteria as they improve the balance of good bacteria in the gut, prevent the growth of undesirable microbes, support proper digestion, enhance the immune system, and improve disease resistance (Islam et al., 2018). Probiotic bacteria that are well-known and utilized as supplements in the beverage industry include *Lactobacillus* and *Bifidobacteria*. Due to exogenous enzymes secreted into the host intestine or endogenous enzymes present in the bacterial cells and released when the acidic environment of the stomach breaks them down, probiotics may also increase the availability of nutrients (Sánchez-Ortiz et al., 2015).

Lactic acid bacteria (LAB) are a group of gram-positive, rod and spherical bacteria. They are non-sporulating, homo fermentative with lactic acid production and hetero fermentative with the capacity to produce mixture of lactic acid, carbon dioxide, acetic acid and ethanol (Nuraida, 2015). LAB are also capable of producing other compounds such as hydrogen peroxide, acetaldehyde, and diacetyl to improve the flavour and texture of fermented foods and also to maintain gut health. LAB, commonly used in food fermentation processes and classified as generally recognized as safe (GRAS), can also be applied in veterinary and medical contexts without posing a significant risk (Hoque et al., 2010). These bacteria are recognized for their role in promoting various beneficial activities within the host gut (De Simone et al., 2023). The phylogenetic range for LAB is broad and includes an extensive variety of species. *Lactobacillus* is a prominent class of bacteria having probiotic characteristics (Jayashree et al., 2010; Heller, 2001; Coeuret et al., 2003). LAB can work antagonistically against other microbes by competing with other organisms for resources and producing various antimicrobials, such as hydrogen peroxide, organic acids, fatty acids, acetoin, and bacteriocins (De Simone et al., 2023; Rocchetti et al., 2021). The significance of probiotics for human health has recently come into focus more clearly. Thus, it is essential to continue looking for strains that show probiotic potential and safety. Since *L. plantarum* must adapt to a variety of environmental pressures, including pH, temperature, bile salt, and osmotic pressure, as well as colonize the intestine and resist harmful bacteria, its safety and probiotic qualities are crucial requirements for its use in humans and animals (Lu et al., 2024).

*Lactiplantibacillus plantarum* is capable of producing bacteriocins, hydrogen peroxide, organic acids, and other compounds that have inhibitory effects on pathogenic bacteria (Zheng et al., 2024). Plantaricin is regarded as a successful alternative to chemical preservatives and antibiotics because of its effectiveness, low toxicity, residues absence, and lack of resistance to antibiotics. Ribosomes produce the bioactive peptide or protein known as plantaricin, which is released into the extracellular space and possesses bacteriostatic or bactericidal properties. Plantaricin is the general term used for bacteriocins that are produced by *Lactiplantibacillus plantarum*. Currently, several strains of *Lactiplantibacillus plantarum* have been found to contain a variety of plantaricin, including A, EF, JK, NC8, S, C, Q7, W, and LpU4 (Zheng et al., 2024). There are notable differences in the number of proteins encoded, the size of the *Lactiplantibacillus plantarum* genome, and the genetic diversity of the gene clusters that produce bacteriocin.

This study reports the isolation and comprehensive genomic characterization of *Lactiplantibacillus plantarum* strain MOVIN, a novel probiotic candidate based on the Type strain genome server (TYGS) obtained from traditionally fermented toddy.

## 2 Methods

### 2.1 Sample collection and isolation

Naturally fermented coconut toddy samples were collected from specific locations within the villages of Paradarami (13.0681° N, 78.9729° E), Palluru (13.0467° N, 79.1365° E), and Bommasamudram (13.0534° N, 79.1266° E), Andhra Pradesh, India. Samples were transferred to the laboratory immediately by maintaining at 4°C for further analysis (Moorthy et al., 2015; Islam et al., 2018). Samples were serially diluted and plated as duplicates on De Man, Rogosa, and Sharpe (MRS) agar using the spread plate technique and incubated at 30°C for 48 h (Moorthy et al., 2015; Vijayabaskar and Somasundaram, 2008). Following incubation, colonies were observed, differentiated based on morphology, and purified by quadrant streak plating (Nath et al., 2021; Vijayabaskar and Somasundaram, 2008).

### 2.2 Assessment of antibacterial activity

The agar well diffusion method was performed to determine the antibacterial activity. Isolates were cultured and incubated in MRS broth for 48 h at 30°C, centrifuged at 8,000 rpm for 20 min and the respective culture supernatants were collected. The activity was tested against *Pseudomonas aeruginosa* (MTCC 2582), *Escherichia coli* (MTCC 443), *Bacillus cereus* (MTCC 121), and *Staphylococcus aureus* (MTCC 3160). Muller Hinton (MH) agar plates were swabbed with indicator strains, which were prepared and incubated overnight, wells were bored, and 100 μL of each culture supernatant was loaded. After that, the plates were incubated at 37°C for 18 h. Ciprofloxacin was used as the antibiotic standard and MRS broth served as a negative control (Moorthy et al., 2015; De Simone et al., 2023; Elayaraja et al., 2014). Zones of inhibition were measured.

### 2.3 Evaluation of probiotic characteristics

#### 2.3.1 Acid, bile and NaCl tolerance

The tolerance of potent isolates to varying pH, NaCl concentrations, and bile concentrations was evaluated. The isolates were cultured in 10 mL of MRS broth adjusted to pH 2, 4, 6, 8, and 10, as well as MRS broth supplemented with NaCl at 2, 4, 6, 8, and 10%, and bile at 0.2, 0.4, 0.6, 0.8, and 1%. A control tube was prepared separately. Each broth was inoculated with 200 μL of seed culture and CFU (colony forming units) of 0th h was identified using the spread plate method for each tube. After that, 200 μL of each culture was loaded onto a 96-well microplate, and using a microplate spectrophotometer, absorbance was measured at 550 nm to evaluate growth under various circumstances for the first hour. Further, the tubes were incubated at 30°C for 3 h and 3rd h CFU was also identified, following that OD was also taken (Sánchez-Ortiz et al., 2015; Kassa et al., 2024; García-Hernández et al., 2016).

#### 2.3.2 Cell surface hydrophobicity

Potent isolates were cultured in MRS broth at 30°C for 24 h. The cells were then collected by centrifugation at 5,000 rpm for 10 min at 4°C, washed twice with phosphate buffered saline (PBS), and resuspended in 6 mL of PBS. The initial absorbance was measured at 600 nm. Subsequently, 1 mL of either p-xylene or chloroform was added to 3 mL of the bacterial suspension, followed by vortexing for approximately 2 min, and then the mixture was allowed to stand undisturbed. The final absorbance at 600 nm was recorded to evaluate cell surface hydrophobicity (Gebre et al., 2023; Nath et al., 2021). The percentage of cell surface hydrophobicity was calculated using,


$$\text{Percentage hydrophobicity}=\frac{{\mathrm{OD}}_{\text{initial}}-{\mathrm{OD}}_{\text{final}}\;}{{\mathrm{OD}}_{\text{initial}}\times 100}$$

#### 2.3.3 Cell auto-aggregation

The auto-aggregation capacity was quantified using cell-to-cell adhesion. Isolates were grown in MRS broth and incubated overnight at 30°C. Subsequently, the cultures were subjected to centrifugation at 5,000 rpm for 10 min at 4°C, followed by two washes with phosphate-buffered saline (PBS). The resulting bacterial pellets were resuspended in 6 mL of PBS, and the initial optical density (OD) was measured at 600 nm. After incubation for 2 h, the final OD at 600 nm was recorded. The auto-aggregation percentage was calculated using the following formula:


$$\text{Percentage auto}-\text{aggregation}=\frac{{\mathrm{OD}}_{\text{initial}}-{\mathrm{OD}}_{\text{final}}\;}{{\mathrm{OD}}_{\text{initial}}\times 100}$$

### 2.4 Safety assessment of potent isolates

#### 2.4.1 Antibiotic susceptibility test

The susceptibility of potent isolates was assessed using the disc diffusion method. The isolates were tested against a panel of antibiotics, including ciprofloxacin, streptomycin, vancomycin, gentamicin, kanamycin, rifampicin, amikacin, amoxicillin, and gatifloxacin. Actively growing cultures were evenly swabbed onto MRS agar plates, onto which antibiotic discs were subsequently applied. The plates were maintained at 4°C for 30 min to facilitate antibiotic diffusion, followed by incubation at 30°C for 48 h. Susceptibility was determined by measuring the diameter of the inhibition zones and categorized as susceptible (≥21 mm), intermediate (16–20 mm), or resistant (≤15 mm) (Nath et al., 2021; Hoque et al., 2010; Kassa et al., 2024).

#### 2.4.2 Haemolytic activity

The isolates were inoculated in blood agar plates (Himedia, India) and incubated at 37°C for 24 h. The appearance of green or clear halos surrounding the bacterial growth on the blood agar was used to determine haemolytic activity (Stojanov et al., 2024). The presence of halos indicated a positive hemolytic reaction, whereas their absence was considered a negative result (Deng et al., 2023).

### 2.5 Antioxidant activity

The antioxidant capacity of the isolates was assessed using the 2,2-diphenyl-1-picrylhydrazyl (DPPH) free radical scavenging assay. Test organisms were grown in freshly prepared MRS broth. Subsequently, 500 μL of the cultured broth was mixed with 1 mL of 0.1 mM DPPH solution, and the mixture was incubated in the dark for 30 min. A control sample was prepared by combining 500 μL of ethanol with 1 mL of DPPH solution. Ascorbic acid was employed as a positive control. The scavenging activity of radicals was determined by measuring the absorbance at 515 nm (Moorthy et al., 2015).

### 2.6 Whole genome analysis of VITVTD-3

Whole Genome Sequencing of sample VITVTD-03 was performed using Oxford Nanopore Technologies (ONT). Nanopore sequencers facilitate the real-time selective sequencing of individual molecules by precisely reversing the voltage across targeted nanopores. *De novo* assembly for the sample was performed using Unicycler assembler (v0.4.8), followed by Ragout scaffolding (Wick et al., 2017; Kolmogorov et al., 2014). Significant BLAST hits functional annotation was performed using BLASTX.

### 2.7 Gene annotation

The final assembled draft genome was used to identify RNA and protein-coding genes in the samples. The gene prediction procedure was carried out using Prokka (v1.12) (Seemann, 2014). The predicted gene sequences from the sample were compared against the NCBI non-redundant protein database (nr) using BLASTX and the Diamond tool (Buchfink et al., 2015). Omicsbox was used for determining the genes’ Gene Ontology (GO) annotations (Götz et al., 2008). GO mapping offers an ontology comprising specific terms that describe gene product attributes. These terms are organized into three main categories: cellular component (CC), molecular function (MF), and biological process (BP). Pathway annotation of the predicted genes was carried out using KAAS (KEGG Automatic Annotation Server), referencing the curated KEGG GENES database (Moriya et al., 2007; Grant et al., 2023).

### 2.8 Detection of secondary metabolites using BAGEL4 and antiSMASH

The secondary metabolite gene clusters produced by VITVTD-03 were analyzed using an antiSMASH bacterial version. In this process, gene clusters are identified using secondary metabolite signatures, and the secondary metabolism clusters of orthologous groups are compared (Zheng et al., 2024; Joishy et al., 2024; Medema et al., 2011). The BAGEL4 bacterial core peptide database was used to search different classes of bacteriocins produced by VIVTD-03 and the number of genes encoding them was identified (Zheng et al., 2024; Zhao et al., 2021; Joishy et al., 2024; van Heel et al., 2018). Additionally, a Comprehensive Antibiotic Resistance Database (CARD) was used to screen the *Lactiplantibacillus plantarum* MOVIN for antimicrobial resistance genes (Joishy et al., 2024; McArthur et al., 2013).

### 2.9 Comparative evaluation of the gene clusters that produce antibiotics

BAGEL4 was utilized to identify the bacteriocin gene clusters in *Lactiplantibacillus plantarum* MOVIN, using *Lactiplantibacillus plantarum* DSM 20174 and *Lactiplantibacillus plantarum* DSM 13273 as reference strains. This approach facilitated comparative analysis of the gene clusters by referencing well-characterized strains. This prediction provides insights into the bacteriocin-producing potential of the MOVIN strain in relation to established *Lactiplantibacillus plantarum* genomes. Investigate the variations across strains according to their Pln genes.

### 2.10 Statistical analysis

All experiments were conducted in triplicates, and the results are expressed as mean ± standard deviation (SD). Statistical significance was evaluated using one-way ANOVA. A *p*-value of <0.05 was considered statistically significant. GraphPad Prism version 8.0.2 (GraphPad Software, United States) was used for all statistical analyses.

## 3 Results

### 3.1 Isolation

**Figure 1:**
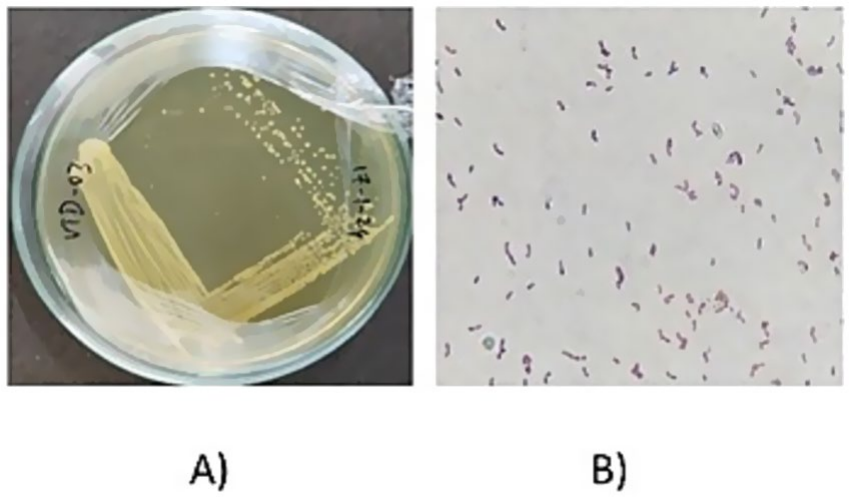
**(A)** Pure culture image of VITVTD-3. **(B)** Gram staining result of VITVTD-3.

The collected samples were serially diluted and the dilutions 10^−2^, 10^−4^, 10^−6^, 10^−8^, and 10^−10^ were plated. Based on the colony morphology, like circular, yellowish white and convex elevation, different isolates were obtained and named as VITVTO 1–7, VITVTD 1–8, and VITVH 1–5. The isolates were streaked on the MRS agar plates for pure culture (Figure 1) and incubated at 30°C for 24 h. The obtained isolates were subjected to primary characterization, out of 20 isolates, 17 isolates were gram-positive, catalase and oxidase negative.

### 3.2 Assessment of antibacterial activity

**Figure 2:**
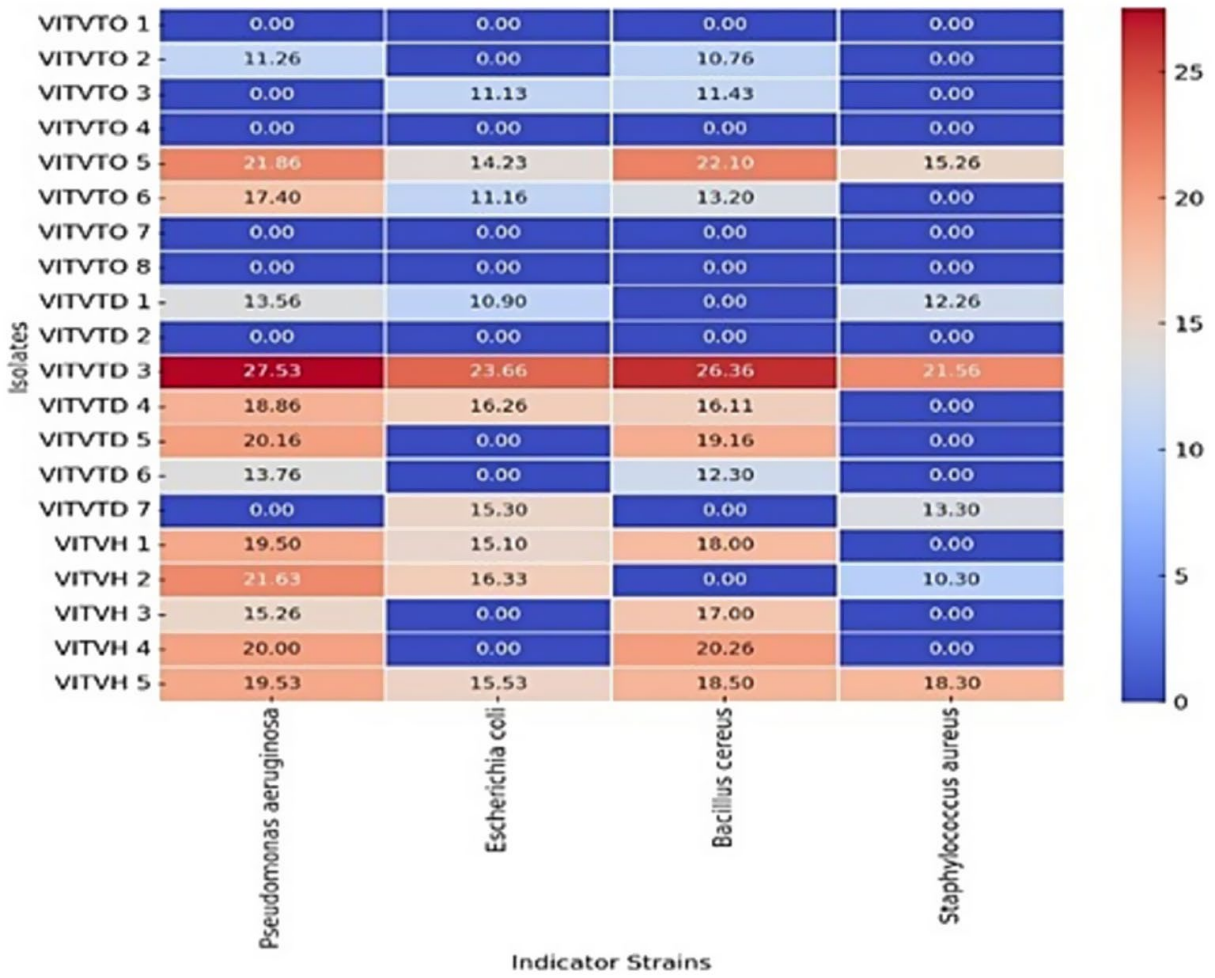
Heat map representation of antibacterial activity.

All the 17 isolates were screened for antibacterial activity by agar well diffusion method. Potent isolates were determined based on the zone of inhibition. Among these, isolates VITVTD3, VITVTO5, and VITVH5 showed significant inhibitory activity against *S. aureus*, *P. aeruginosa*, *E. coli*, and *B. cereus* (Figure 2). The largest zone of inhibition of 27.53 mm was demonstrated by VITVTD-3, which indicated high antibacterial activity, suggesting its broad-spectrum antibacterial potential. The zones were compared with the Kirby-Bauer method, which shows VITVTD-3 is sensitive to all the indicator strains, VITVTO-5 is sensitive to two and intermediate for the other two and VITVH-5 is intermediate for all the indicator strains.

### 3.3 Evaluation of probiotic characteristics

#### 3.3.1 Acid, NaCl, and bile tolerance

**Figure 3:**
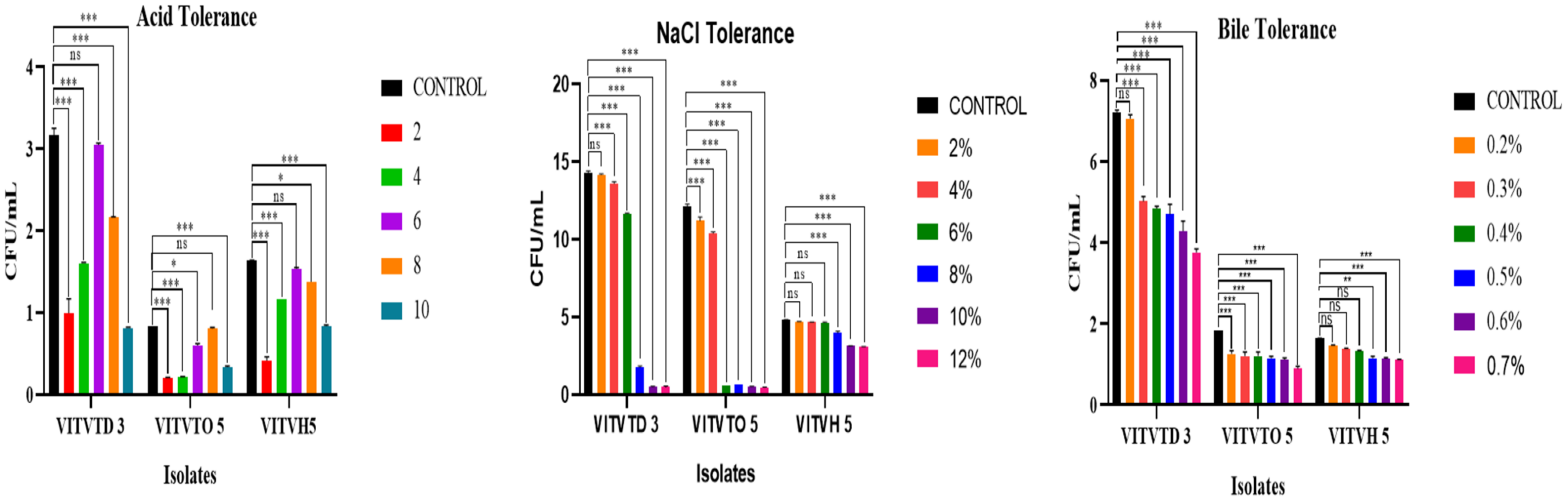
**(A)** Acid tolerance. **(B)** NaCl tolerance. **(C)** Bile tolerance results of isolates VITVTD-3, VITVTO-5, and VITVH-5.

The effects of pH, NaCl, and bile concentration on the growth of the tested isolates are illustrated in Figure 3. Figure 3A indicates that all isolates were capable of growing in low pH; comparatively, VITVTD-3 has more CFU counts than the other two isolates. Maximum growth was obtained in VITVTD-3 with 3 × 10^7^ CFU/mL at pH 6. The decrease in CFU was observed by reducing the pH below and above the control (pH 7). Figure 3B indicates that the growth gradually decreases with the increase of the concentration of NaCl. Mostly, the isolates were able to grow up to 6–8%. VITVTD-3 has high CFU, making it more potent comparatively. The control broth of VITVTD-3 has 15 × 10^7^ CFU/mL. From Figure 3C, the growth of all the isolates decreases with increasing concentration of bile salts. The changes in the growth in different concentrations are shown as significant compared with the control. The potent isolate VITVTD-3 has >7 × 10^7^ CFU/mL at 0.2% of bile concentration and the growth significantly decreases with an increase in the concentration.

#### 3.3.2 Cell surface hydrophobicity

The isolates VITVTD-3, VITVTO-5, and VITVH-5 exhibited 59.59, 61.09 and 35.79% cell surface hydrophobicity, respectively. Two isolates, VITVTD-3 and VITVTO-5, exhibited inhibition zones exceeding the threshold percentage required for effective hydrophobicity. It is evident that the isolates possess the potential to adhere to the intestinal mucosa which was inferred indirectly through their cell surface hydrophobicity (Gebre et al., 2023).

#### 3.3.3 Cell auto-aggregation

The auto-aggregation assay revealed that two isolates, VITVTO-5 and VITVH-5, exhibited relatively low auto-aggregation capacities, with values below 20%. This suggests a limited ability of these strains to self-aggregate under the tested conditions, which may affect their potential to form stable colonizing populations in the gastrointestinal tract. In contrast, the isolate VITVTD-3 demonstrated a notably higher auto-aggregation capacity of 27%, indicating a stronger tendency for cell-to-cell adherence. Such a characteristic is often associated with better gut colonization potential, biofilm formation, and exclusion of pathogenic bacteria by competitive adherence (see Table 1).

**Table 1:** The percentage of hydrophobicity and auto-aggregation.

Activity isolates	Cell surface hydrophobicity	Auto-aggregation
VITVTD-3	59.5921	27.884
VITVTO-5	61.09422	10.6383
VITVH-5	35.79148	18.6904

### 3.4 Safety assessment

#### 3.4.1 Antibiotic susceptibility test

**Figure 4:**
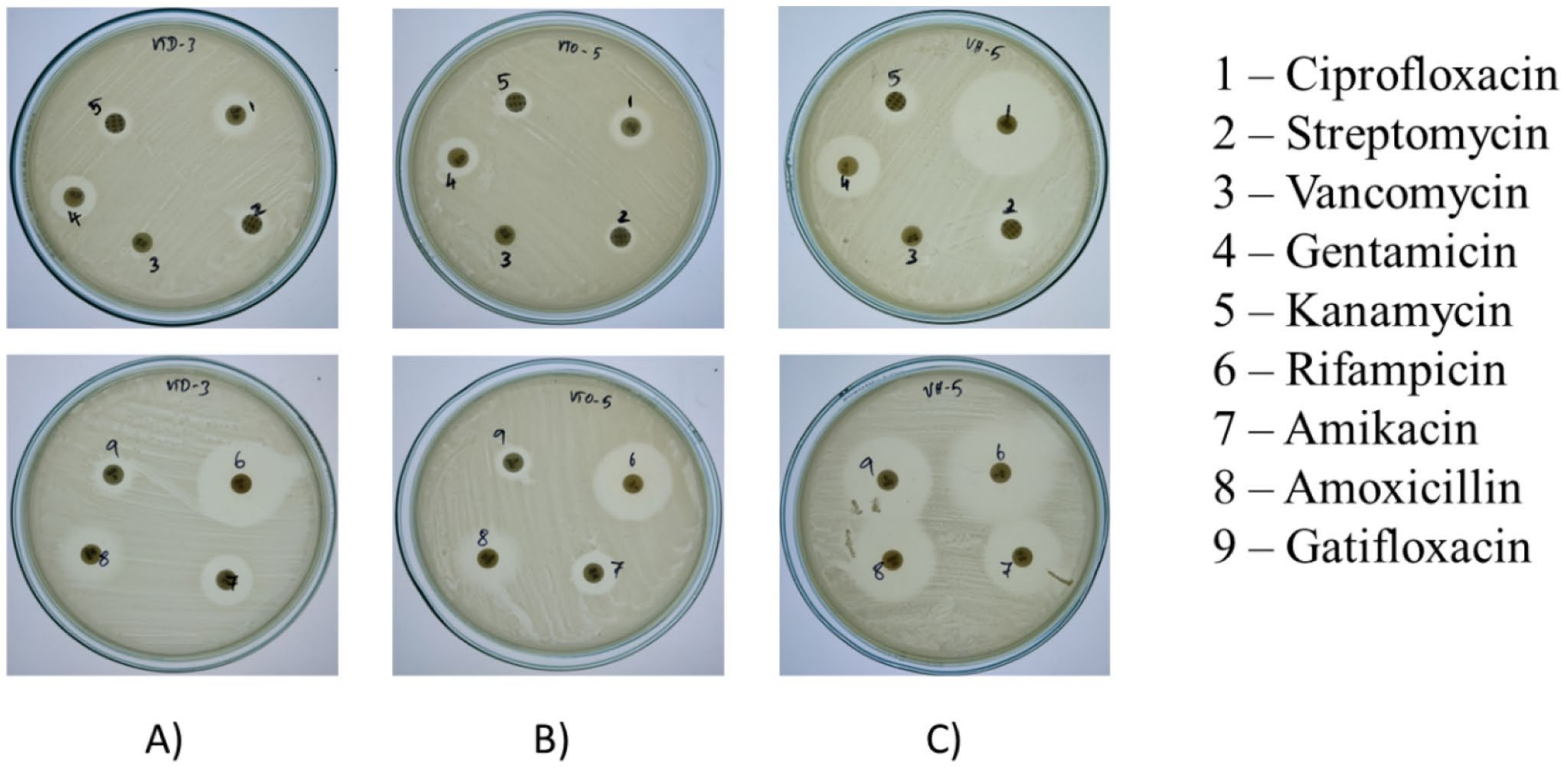
Zone of antibiotic susceptibility test for **(A)** VITVTD-3, **(B)** VITVTO-5, and **(C)** VITVH-5.

Results indicate variable resistance and susceptibility patterns among the isolates VITVTD-3 (Figure 4A), VITVTO-5 (Figure 4B), and VITVH-5 (Figure 4C). VITVH-5 exhibited the highest susceptibility, being sensitive to ciprofloxacin, gentamicin, rifampicin, amikacin, amoxicillin, and gatifloxacin, suggesting its potential as a safe probiotic candidate. VITVTD-3 showed moderate antibiotic tolerance, with susceptibility to gentamicin, rifampicin, and amikacin and intermediate resistance to ciprofloxacin, amoxicillin, and gatifloxacin, indicating that it may also be considered a probiotic strain. VITVTO-5 displayed the highest resistance, being resistant to ciprofloxacin, streptomycin, vancomycin, and kanamycin, which may limit its probiotic potential. The complete resistance to vancomycin across all isolates raises concerns about possible intrinsic resistance mechanisms, warranting further molecular analysis. The zones were measured and sizes were given in Table 2.

**Table 2:** Antibiotic susceptibility test zone size (in mm).

Antibiotic	Breakpoints (S/I/R) mm	VTD 3 (mm)	VTO 5 (mm)	VH 5 (mm)
Ciprofloxacin	S ≥ 21, I = 16–20, R ≤ 15	**I** (16.33)	**R** (14.23)	**S** (35.76)
Streptomycin	S ≥ 15, I = 12–14, R ≤ 11	**I** (11.5)	**R** (10.43)	**S** (13.96)
Vancomycin	S ≥ 17, I = 15–16, R ≤ 14	**R** (0)	**R** (0)	**R** (0)
Gentamicin	S ≥ 15, I = 13–14, R ≤ 12	**S** (14.96)	**I** (14.2)	**S** (22)
Kanamycin	S ≥ 18, I = 14–17, R ≤ 13	**R** (11.63)	**R** (11.7)	**I** (13.3)
Rifampicin	S ≥ 20, I = 17–19, R ≤ 16	**S** (28.46)	**S** (27.53)	**S** (33.53)
Amikacin	S ≥ 17, I = 15–16, R ≤ 14	**S** (16.73)	**I** (14.9)	**S** (25.56)
Amoxicillin	S ≥ 20, I = 14–19, R ≤ 13	**I** (16.93)	**I** (17.3)	**S** (28.53)
Gatifloxacin	S ≥ 18, I = 14–17, R ≤ 13	**I** (14.3)	**I** (12)	**S** (29.53)

The bold values: R stand for Resistant; I stand for Intermediate; and S stand for Susceptible. It indicates the susceptibility nature of the lactic acid bacteria when administered with the antibiotics.

#### 3.4.2 Haemolytic activity

**Figure 5:**
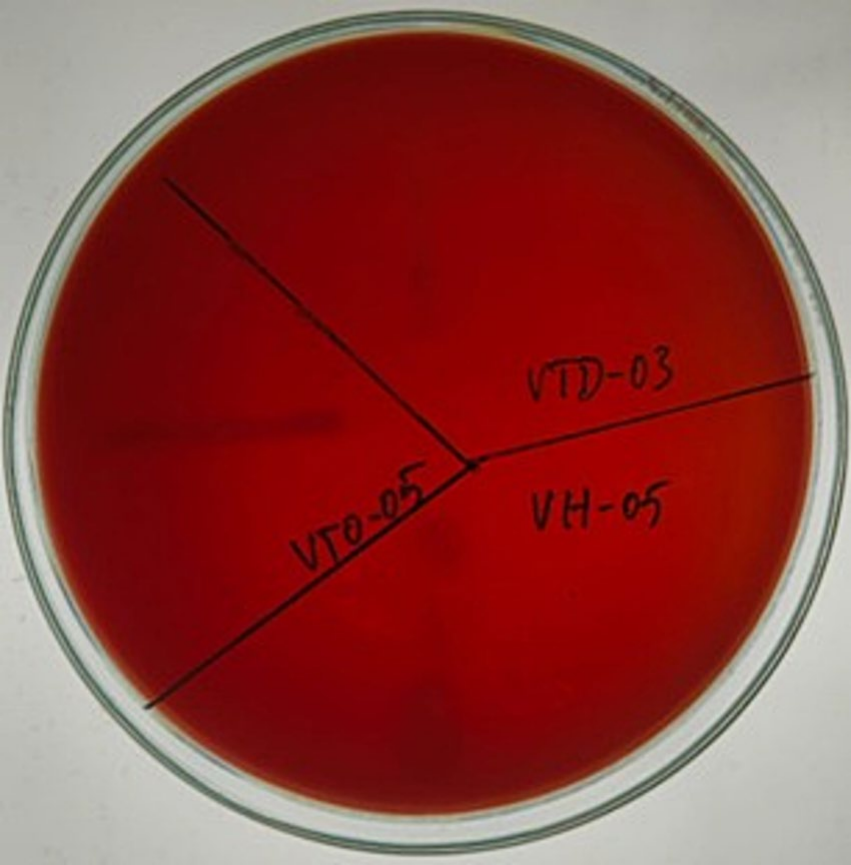
Assessment of haemolytic activity of isolates VITVTD-3, VITVTO-5, and VITVH-5.

The blood agar plates of the three potent isolates were observed for the haemolysis after incubation. The obtained results confirmed that the isolates subjected to the activity did not exhibit haemolytic activity (Figure 5).

### 3.5 Antioxidant activity

DPPH assay shows the result of the free radical scavenging property of the test isolates. The isolates VITVTD-3, VITVTO-5, and VITVH-5 showed 58.8, 53.3 and 54.9% of scavenging, respectively. The positive control, ascorbic acid (100 μg/mL), showed 73.8% of scavenging of free radicals. The test samples showed a considerable percentage of scavenging when compared to the positive control used (see Table 3).

**Table 3:** Antioxidant activity.

S. No.	Sample	Absorbance (OD-515 nm)	Percentage of activity
1	VITVTD-3	0.934 ± 0.001	58.8
2	VITVTO-5	1.057 ± 0.0015	53.3
3	VITVH-5	1.02 ± 0.002	54.9
4	Ascorbic acid	0.594 ± 0.0005	73.8

### 3.6 Genomic characterization of VITVTD-3

**Figure 6:**
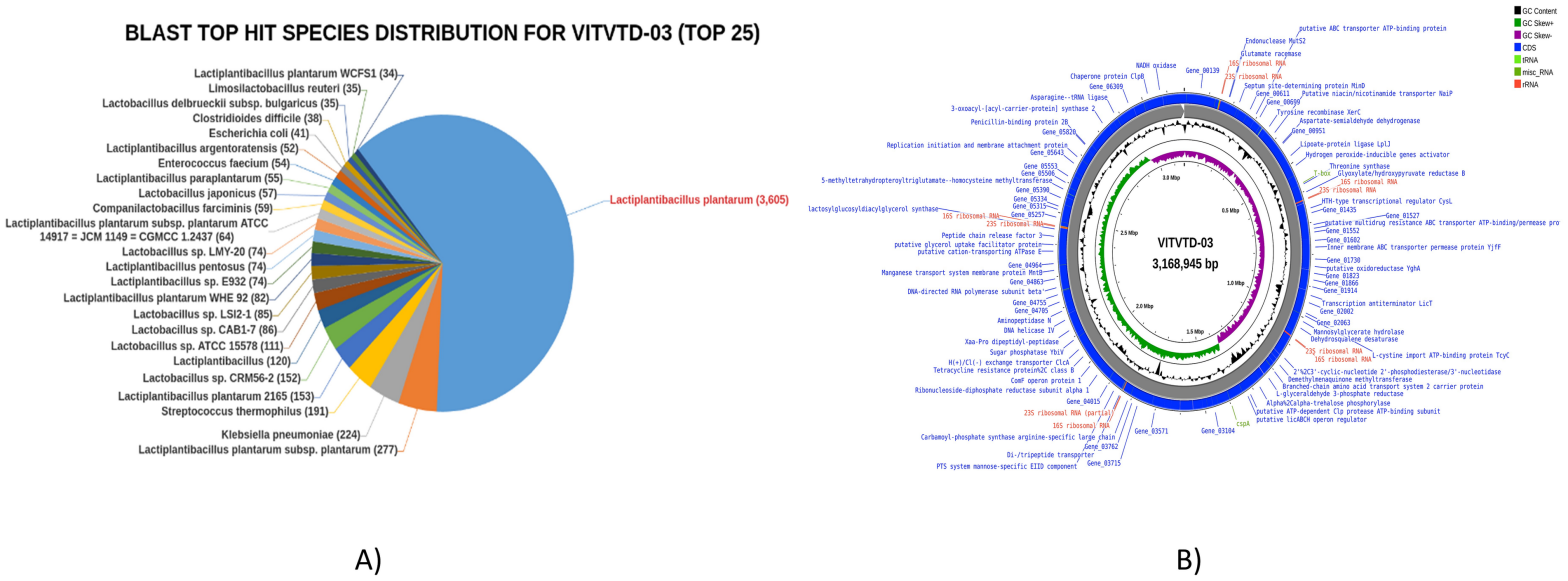
**(A)** Blast top hits species for *Lactiplantibacillus plantarum* MOVIN. **(B)** Circle map of *Lactiplantibacillus plantarum* MOVIN.

The whole genome of VITVTD-03 was successfully sequenced and identified as *Lactiplantibacillus plantarum*, yielding 3.36 Gb of raw sequencing data. The genome size was determined to be 3.16 Mb with an average GC content of 44.4%. Analysis of the top BLAST hits revealed that the majority of the genes showed high homology with *Lactiplantibacillus plantarum*, as illustrated in Figure 6A. The genome comprised a total of 6,779 genes, including 6,677 protein-coding sequences, 15 ribosomal RNA genes, and 47 transfer RNA genes. A circular genome map illustrating gene locations, GC content, and other genomic features was generated using the Proksee tool (Figure 6B). Additionally, the genome sequence of *Lactiplantibacillus plantarum* strain MOVIN has been deposited in the NCBI GenBank database under accession number CP185291.

### 3.7 Gene annotation

**Figure 7:**
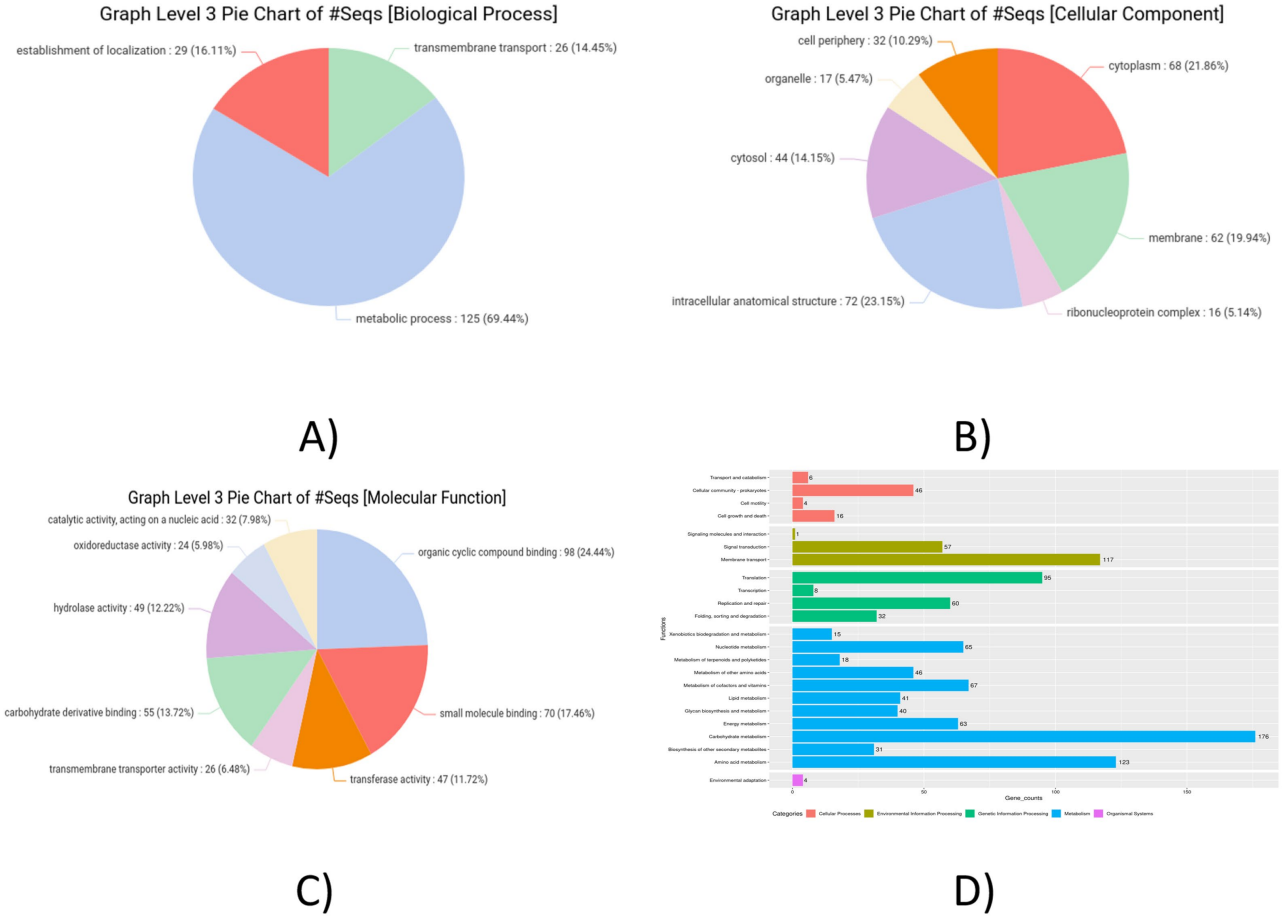
Graph level 3 pie chart. **(A)** Biological process. **(B)** Cellular component. **(C)** Molecular functions. **(D)** Pathway analysis KAAS graph of *Lactiplantibacillus plantarum* MOVIN.

The distribution of Gene Ontology terms across the categories—BP, CC, and MF was obtained and the result analysis was represented in pie chart formats (Figure 7). Most of the genes were found to be associated with the GO main domain “Molecular Function.” Most of the genes were linked to the metabolism of amino acids, translation, carbohydrates, membrane transport, signal transduction, cofactor and lipid metabolism, production of secondary metabolites, and cell motility, according to the functional annotation study conducted using KEGG. Figure 7 shows the metabolic pathways of genes which were predicted using the KEGG automated annotation service, KAAS.

### 3.8 Detection of secondary metabolites using BAGEL4 and antiSMASH

**Figure 8:**
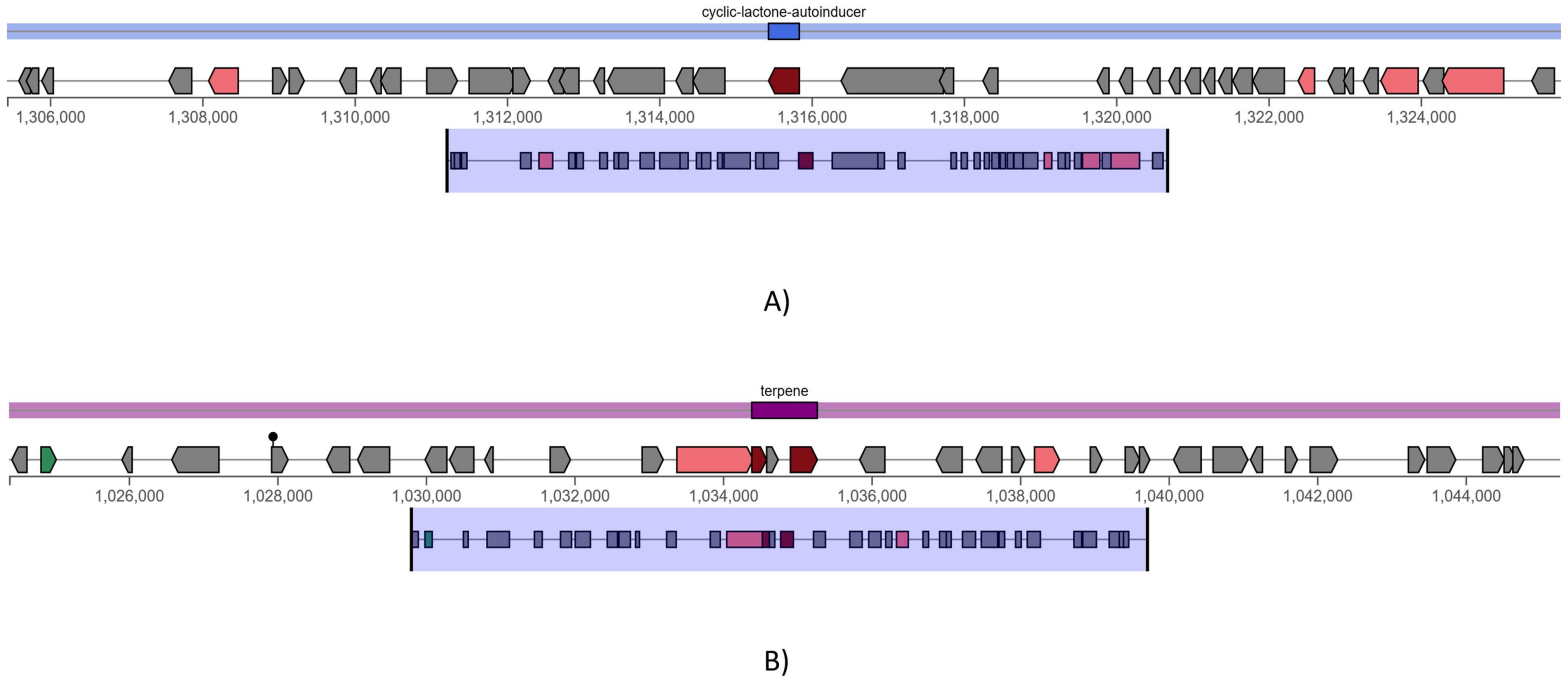
antiSMASH predicted secondary metabolite production pathways, **(A)** cyclic-lactone-autoinducer and **(B)** terpene.

Two potential pathways were found using antiSMASH secondary metabolite detection software program. One is terpene (Figure 8B), which complements bacteriocins by offering additional antimicrobial activity metabolites and cyclic-lactone-autoinducer (Figure 8A), which indicates quorum-sensing regulation of bacteriocin gene clusters. Additionally, the genome was examined for genes linked to antimicrobial resistance (AMR). Glycopeptide resistance gene clusters were detected, which were associated with resistance to glycopeptide antibiotics like vancomycin and teicoplanin. This is primarily due to the modification of the van operon genes. Using the iProbiotics server, the strain was confirmed as a probiotic organism with a 99.97% match.

### 3.9 Comparative evaluation of the gene clusters that produce antibiotics

**Figure 9:**
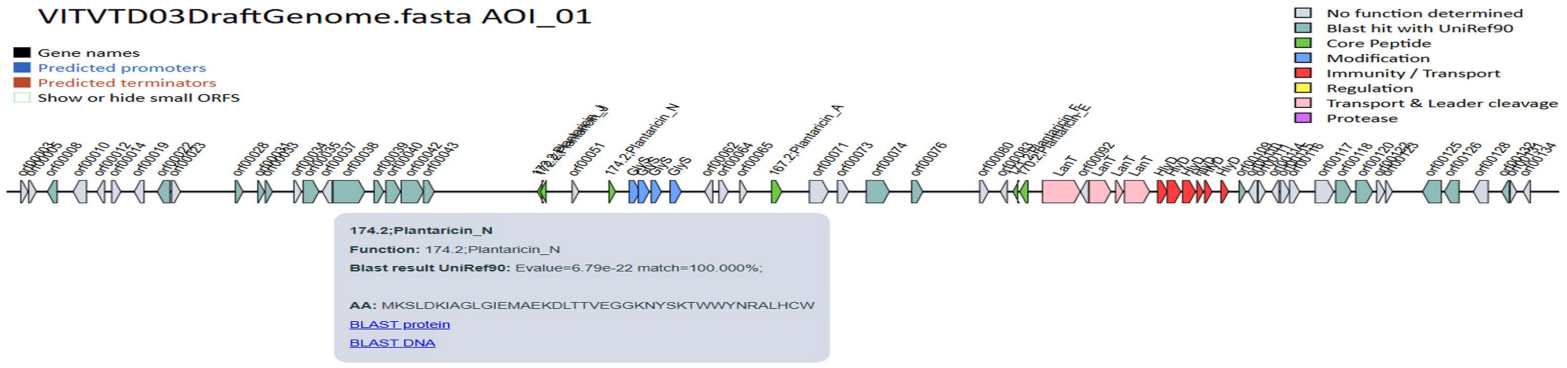
BAGEL4 predicted bacteriocin-encoding genes.

**Figure 10:**
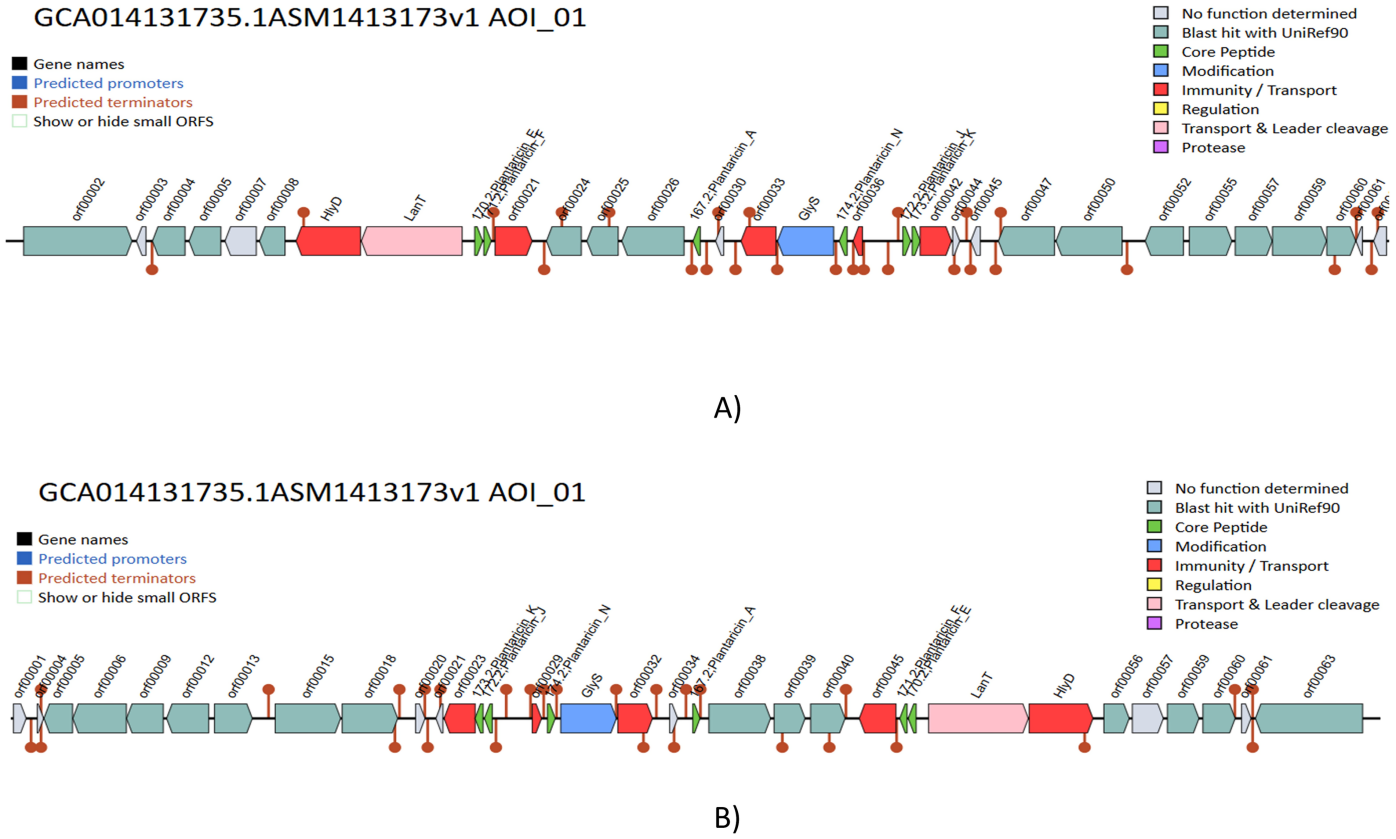
Comparative evaluation of gene clusters that produce bacteriocin in various *Lactiplantibacillus plantarum*, **(A)** *Lactiplantibacillus plantarum* DSM 20174 and **(B)** *Lactiplantibacillus plantarum* DSM 13273.

The genome was screened for putative bacteriocin operons and identified various classes of bacteriocins. The significant similarity was observed with the plantaricin family, which includes plantaricin-J (match-95.45%, *E*-value-3.47 × 10^−13^), plantaricin-N (match-100%, *E*-value-6.79 × 10^−22^), plantaricin-A (match-100%, *E*-value-5.17 × 10^−10^), plantaricin-F (match-100%, *E*-value-3.145 × 10^−15^) and plantaricin-E (match-87.87%, *E*-value-5.61 × 10^−18^), which is shown in Figure 9. The comparative analysis of genes to their corresponding bacteriocins was analyzed with similar strains *L. plantarum* DSM 20174 and *L. plantarum* DSM 13273 based on type strain genome server results shown in Figures 10A,B, respectively. By comparing the bacteriocin gene cluster of VITVTD-03 with the pairwise compared strains, it showed a high similarity of bacteriocin gene cluster presence.

## 4 Discussion

*L. plantarum* is used extensively in the food industry and exhibits resistance to acidic environments, extending the shelf life of the food products (Joishy et al., 2024). In this study, a total of 20 isolates (VITVTO 1–8, VITVTD 1–7, and VITVH 1–5) were obtained from different fermented toddy samples from different regions. Based on the preliminary LAB characterization, like Gram staining, catalase and oxidase test, 17 strains were selected and further subjected for antibacterial activity. The isolates VITVTD-3, VITVTO-5, and VITVH-5 have exhibited inhibitory activity against all four food-borne pathogens, like *P. aeruginosa MTCC 2582*, *E. coli MTCC 443*, *B. cereus MTCC 121*, and *S. aureus MTCC 3160*. The zone of inhibition (mm) for the cell-free supernatants of VITVTD-3, VITVTO-5 and VITVH-5 were found to be 27.53 ± 0.29, 21.86 ± 0.35 and 19.53 ± 0.26 against *Pseudomonas aeruginosa MTCC 2582*, 23.66 ± 0.24, 14.23 ± 0.33 and 15.53 ± 0.14 against *Escherichia coli MTCC 443*, 26.36 ± 0.23, 22.1 ± 0.1 and 18.5 ± 0.17 against *Bacillus cereus MTCC 121* and 21.56 ± 0.34, 15.26 ± 0.17 and 18.3 ± 0.15 against *Staphylococcus aureus MTCC 3160*, *respectively*. A similar study conducted by Joishy et al. (2024), *Lactiplantibacillus plantarum* strains MKTJ24 and MKTJ23 produced <15 mm of inhibitory zone against *Staphylococcus aureus*. Comparatively VITVTD-3, VITVTO-5, and VITVH-5 produced larger zone of inhibition against all four pathogens particularly against *Staphylococcus aureus*. Hence it was evident that these three strains could effectively inhibit the food borne pathogens and considered for further studies (Vijayabaskar and Somasundaram, 2008; Verschuere et al., 2000).

Furthermore, to determine probiotic properties, assays were conducted for these three isolates. The most important function of probiotic strains is their capacity to withstand an acidic environment, with the stomach pH level as low as 1.5–3. Based on the probiotic study conducted for VITVTD-3, VITVTO-5, and VITVH-5, the capability for acid, bile and NaCl tolerance were verified (Lu et al., 2024; Joishy et al., 2024; Lee et al., 2024). The maximum growth was observed for VITVTD-3 at pH 6 with 3 × 10^7^ CFU/mL and even at pH 2 the significant growth was observed with 1 × 10^7^ CFU/mL. The study conducted on *Weissella confusa* strain GCC_19R1 showed significant growth at pH 3 (Nath et al., 2021). In addition, *L. plantarum* EL2, the strain, showed significant growth at pH 2 (Zheng et al., 2024) and out of three strains used in this study, VITVTD-3, showed significant growth at pH 2. For NaCl tolerance, reduction of growth was observed in increase in NaCl concentration more than 6–8%. A key characteristic of probiotic bacteria is their capacity to adhere to epithelial cells is known as cell surface hydrophobicity. In the current study, VITVTD-3, VITVTO-5, and VITVH-5 showed hydrophobicity of 59.59, 61.1 and 35.79%, respectively (Chen et al., 2023; de Oliveira Rocha et al., 2024). Antibiotic-susceptible probiotic strains have a crucial role in limiting the horizontal spread of antibiotic resistance. Clinical therapy may severely be limited by the extended consumption of resistant lactic acid bacteria, which is the reason *lactobacillus* resistance is a major area of focus (Lu et al., 2024). According to earlier research, *Pediococci* are resistant to the genes for streptomycin, tetracycline, vancomycin, and ciprofloxacin, but *Lactobacilli* are sensitive to these antibiotics (Danielsen et al., 2007). All the three isolates were found to be susceptible to antibiotics except vancomycin. The haemolytic assay revealed that all the three strains were non haemolytic in nature. The antioxidant assay measures the ability of a substance to neutralize free radicals and prevent oxidative damage. The radical scavenging ability of the isolates was evaluated using DPPH method. The results revealed that the isolates VITVTD-3, VITVTO-5, and VITVH-5 have 58.8, 53.3 and 54.9% of scavenging, respectively.

Based on the preliminary results obtained, VITVTD3 was considered to be more potent than VITVTO5 and VITVH5. Hence, VITVTD-3 was subjected to whole genome sequencing for strain-level identification and identified as *Lactiplantibacillus plantarum*. It was further analysed through type strain genome server (TYGS) and found to be novel. Hence, it was named as *Lactiplantibacillus plantarum* MOVIN. The genome results showed that the genome size of 3.14 Mb with 6,779 predicted genes is similar to that of most *Lactiplantibacillus plantarum* genomes. From the gene annotation of *Lactiplantibacillus plantarum* MOVIN, a heat shock protein htpX and a small heat shock protein HP15 were identified. HcA, a transcriptional regulator of dnak and groE, was also identified. Additionally, GRPE and ATP-dependent Clp protease molecular chaperone proteins were also identified. The presence of all these genes indicate that this strain exhibits a high level of temperature resistance. Also, a possible cold shock protein called cspA has been discovered. A multicomponent binding ABC transport system (opuA and opuBD) is encoded by the *L. plantarum* MOVIN genome to handle possible prolonged osmotic stress in the gastrointestinal tract. A similar genome was also identified in a study conducted by Lu et al. (2024) on *Lactiplantibacillus plantarum* HYY-DB9; additionally, opuBB was identified in *Lactiplantibacillus plantarum* MOVIN.

The entire genome of VITVTD-03 was sequenced and characterized. Genomic analysis of *Lactiplantibacillus plantarum* MOVIN revealed the presence of several key proteins, including Na^+^/H^+^ antiporters. These proteins play a critical role in acidic environments by utilizing cellular ATP to expel excess H^+^ ions from the cells. This mechanism helps maintain intracellular pH stability, thereby enhancing the cells’ ability to adapt and survive in acidic conditions (Wu et al., 2023). Two essential components that help probiotics to survive in the severe conditions of the intestines are bile salt hydrolase and transporters. *Lactiplantibacillus plantarum* MOVIN exhibits tolerance to 0.3 and 0.7% bile salt up to 3 h, attributed to the presence of choloylglycine hydrolases, which function as bile salt hydrolases in its genome. Additionally, bile salt resistance is supported by an efflux system involving multidrug resistance transporters, including members of the ABC transporter family and the major facilitator superfamily (MFS) gene, both identified within the genome of *L. plantarum* MOVIN. Similarly, *Lactiplantibacillus plantarum* MKTJ24 possesses comparable genetic components contributing to bile salt tolerance (Wu et al., 2023). The average hydrophobicity of *Lactiplantibacillus plantarum* MOVIN was 59.59%, greater than the 42 and 42.96% found in similar research with *Lactiplantibacillus plantarum* MKTJ23 and *Lactiplantibacillus plantarum* HYY-DB9, respectively (Lu et al., 2024; Joishy et al., 2024). Colonization and pathogen exclusion, which impact host immunity, depend on the probiotic’s capacity to stick to the intestinal epithelium (Ayyash et al., 2018). According to a genomic study, *Lactiplantibacillus plantarum* MOVIN was shown to have stronger adhesion qualities. The presence of fibronectin-binding proteins (fbp), enolases, sortase (srtA), LPXTG-anchored proteins, lspA, and exoA could potentially supports this phenomenon.

The genome annotation of *L. plantarum* MOVIN identified genome clusters like terpene and cyclic lactone autoinducer peptides by implementing antiSMASH 7.0, which enhances the antimicrobial potential of this strain. By employing BAGEL4 (van Heel et al., 2018), it was identified the presence of plnA, plnEF, plnJ and plnN genes, which encode bacteriocin (Joishy et al., 2024). All of these genes were proven to have strong antibacterial properties. According to earlier studies, bacteriocins are frequently utilized in food preservation because they inhibit the growth of diseases and putrefactive bacteria. Bacteriocins have a wide pH range, which helps to prolong the shelf life of certain products (Joishy et al., 2024). Since *Lactiplantibacillus plantarum* ULAG24 has plantaricin genes, it was antagonistic to foodborne pathogens (Oguntoyinbo and Narbad, 2015). Similarly, it has been found that *Lactiplantibacillus plantarum* Q7 and *L. plantarum* F3-2, which produce bacteriocin, decrease the harmful bacteria in mice’s guts (Bu et al., 2023). The gene function annotation of *Lactiplantibacillus plantarum* revealed an ATPase component, the ABC-type antimicrobial peptide transport system, which facilitates the passage of antimicrobial peptides across the membrane. Using the Comprehensive Antimicrobial Resistance Gene Database (CARD), the vancomycin resistance gene (glycopeptide resistance gene cluster), which has already been reported in other LAB species such *Lactobacilli*, *Leuconostoc*, and *Pediococci*, was also detected by genomic study of *Lactiplantibacillus plantarum* MOVIN (Joishy et al., 2024; Hamilton-Miller and Shah, 1998; Simpson et al., 1988). Further investigation revealed that the strain MOVIN’s reported vancomycin resistance determinants are inherent to *Lactiplantibacillus plantarum*, a characteristic that is frequently observed and thought to be non-transferable (Zheng et al., 2020). Its probiotic effectiveness was further supported by iProbiotics study, which verified its categorization with 99.97% accuracy. This study establishes a basis for future research into the underlying mechanisms responsible for the probiotic properties of *Lactiplantibacillus plantarum* MOVIN. The findings pave the way for the development of functional foods and support further exploration and application of *Lactiplantibacillus plantarum* MOVIN as potential probiotics.

## 5 Conclusion

In conclusion, *Lactiplantibacillus plantarum* MOVIN was successfully discovered as a powerful probiotic candidate with bacteriocin production, adhesion ability, stress tolerance, and antioxidant potential. Strong adhesion potential, which is necessary for gut colonization, high survival rates in acidic pH and bile salt environments, and remarkable antibacterial activities against pathogenic bacteria were all displayed by the isolate. Its usefulness as a probiotic was ensured by safety evaluations, which verified its non-haemolytic nature and sensitivity to the majority of antibiotics. Its antibacterial activity was subsequently confirmed by whole-genome sequencing, which identified genes linked to bacteriocin production, stress response, and carbohydrate metabolism, including many operons of the plantaricin family. Its probiotic effectiveness was further supported by the iProbiotics study, which verified its categorization with 99.97% accuracy. These findings suggest that *L. plantarum* MOVIN holds significant potential for therapeutic applications and functional food development. Future research will primarily focus on *in vivo* validation and the formulation process to enable its commercialization as a probiotic product.

## Data Availability

The original contributions presented in the study are publicly available. This data can be found here: https://www.ncbi.nlm.nih.gov, accession number CP185291.
